# Clinical application of ultrasound-guided plastic cannulae for initial AVF puncture in uraemic patients

**DOI:** 10.1093/ckj/sfag040

**Published:** 2026-02-12

**Authors:** Qiu-Han Chen, Jian-Ying Tang, Lin-Juan Pu, Jun-Chen Li

**Affiliations:** Department of Nephrology, University-Town Hospital of Chongqing Medical University, Chongqing,China; Department of Nephrology, University-Town Hospital of Chongqing Medical University, Chongqing,China; Department of Nephrology, University-Town Hospital of Chongqing Medical University, Chongqing,China; Department of Nephrology, University-Town Hospital of Chongqing Medical University, Chongqing,China

To the Editor,

We conducted a study on the application effect of plastic cannulae for dialysis combined with ultrasound guidance in the initial puncture of arteriovenous fistulas (AVFs) in uraemic patients. The main findings are reported below.

AVFs are the ‘lifeline’ for maintenance haemodialysis patients, and the puncture technique directly affects dialysis efficacy and patients’ quality of life [1]. Traditional puncture methods rely on nurses’ tactile perception, often leading to puncture failure and vascular access complications. Studies have shown that AVF puncture remains a technical challenge even for experienced dialysis nurses [2]. The introduction of ultrasound-guided technology provides a new solution to this challenge, as it can clearly visualize the AVF outflow vein and assist in puncture planning. Meanwhile, traditional dialysis needles may cause various complications and patient discomfort [3]. Plastic cannulae, as an alternative, have shown advantages in improving puncture efficiency and patient comfort. However, there is still a lack of systematic research on the application effect of indwelling needles combined with ultrasound-guided technology in the initial puncture for an AVF (Figs. 1 and 2).

**Figure 1:**
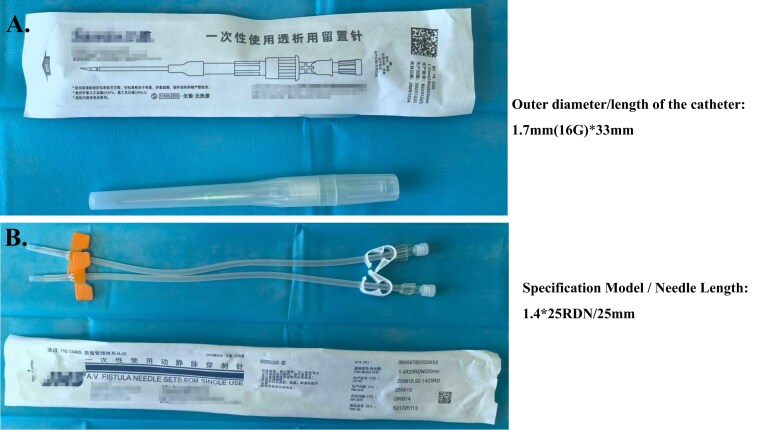
**(A)** For plastic cannulae, outer diameter/length of the catheter: 1.7 mm (16 G)*33 mm. **(B)** For traditional steel needles, specification model/needle length: 1.4*25RDN/25 mm.

**Figure 2:**
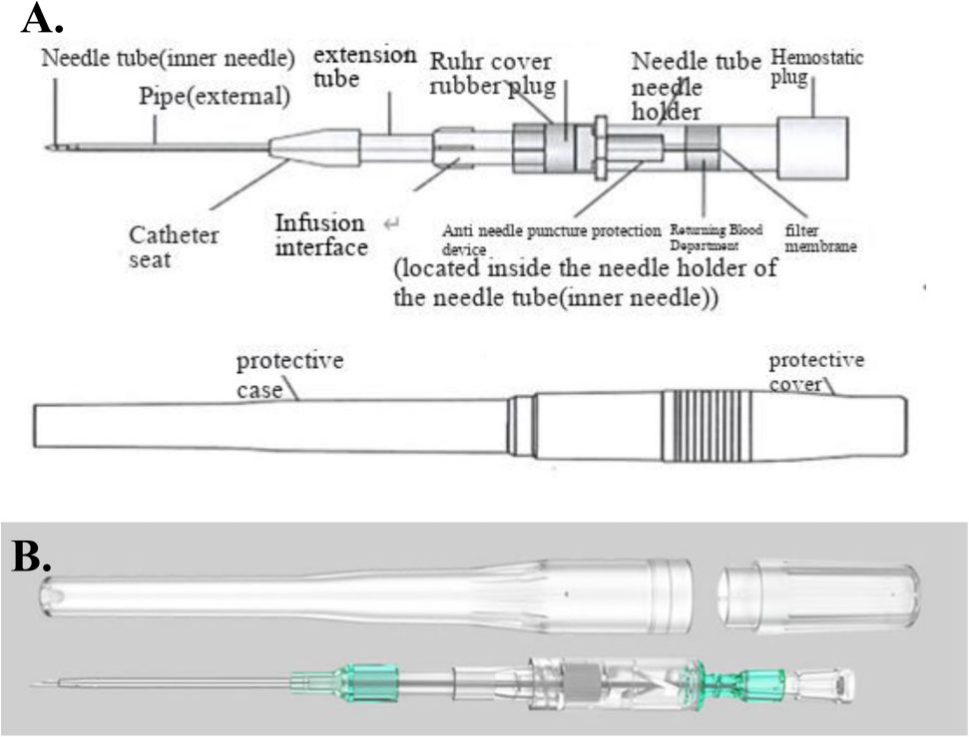
Schematic diagram of the structure of a typical blood dialysis plastic cannulae. **(A)** Structural analysis of plastic cannulae. **(B)** The appearance of the plastic cannulae packaging after opening. Plastic cannulae instructions for use: After disinfection, similar to regular indwelling needle puncture, observe the return of blood at the end of the needle. If there is blood return, it indicates that the steel needle has penetrated the blood vessel. Push the hose first and then retract the steel needle to ensure a smoother passage of the hose into the blood vessel.

A randomized controlled trial was designed, enrolling 40 uraemic patients with newly established AVFs (fistula maturity 4–8 weeks post-operation, ultrasound-measured vessel diameter ≥2.5 mm, blood flow ≥500 ml/min). They were randomly divided into an experimental group (plastic cannulae + ultrasound guidance, *n* = 20) and a control group (traditional steel needle + palpation localization, *n* = 20). Both groups received standardized nursing interventions: pre-puncture ultrasound/palpation assessment of vascular conditions; standardized puncture operation (20°–30° needle insertion, parallel advancement after blood return); U-shaped tape combined with bridge-arch fixation; and dynamic monitoring during the indwelling period (≤4 hours). Primary outcome measures included first-attempt success rate, operation time, complication rate and pain score (numeric rating scale). All procedures were performed by specially trained medical staff.

The results showed the experimental group had a significantly higher first-attempt success rate (90% versus 70%), a shorter average operation time (4.95 ± 0.29 min versus 7.60 ± 0.37 min; *P* < .05), a significantly lower incidence of minor oozing (10% versus 25%), no cases of haematoma or catheter dislodgement and significantly better pain scores (1.25 ± 0.12 versus 1.90 ± 0.23; *P* < .05) (Table 1).

**Table 1:** Comparison of puncture outcomes.

Outcome	PC group	TSN group	*P*-value
Success rate, %	90	70	–
Procedure time (minutes), mean ± SD	4.95 ± 0.29	7.60 ± 0.37	<.001
Bleeding, %	10	25	–
Pain score, mean ± SD	1.25 ± 0.12	1.90 ± 0.23	<.05

PC: plastic cannulae; TSN: traditional steel needle.

This study is the first to systematically evaluate the application value of plastic cannulae combined with ultrasound guidance in initial AVF puncture. The findings support the clinical promotion of this combined technique. The advantages of ultrasound guidance in AVF puncture have been confirmed by multiple studies. It provides real-time visualization of vessel course and blood flow, avoiding complications caused by blind puncture. Particularly for complex AVFs or deeply located vessels, ultrasound guidance significantly improves puncture success rates. Our study further demonstrates that combining ultrasound guidance with plastic cannulae technology produces a synergistic effect, yielding better clinical outcomes.

The role of plastic cannulae in reducing puncture-related pain is particularly notable [4]. Compared with traditional metal dialysis needles, plastic cannulae cause less tissue damage and patient discomfort [5], as our study showed significantly lower pain scores in the combined-technology group.

However, this study has the following limitations: the single-centre design may limit generalizability, the sample size was relatively small and there was a short follow-up duration without long-term AVF function assessment. Future multicentre, large-sample, long-term follow-up studies are needed to validate these findings.

In conclusion, the combined use of plastic cannulae for dialysis and ultrasound guidance demonstrates significant advantages in the initial puncture of AVFs in uraemic patients, including higher puncture success rates, fewer complications, reduced patient pain, shorter operation time and improved long-term fistula function. This combined technique is worthy of clinical promotion, particularly for initial AVF puncture and patients with complex vascular access.

## Data Availability

Data are available in the letter.
